# PILOT STUDY OF A VIRTUAL ART KIT PROGRAM FOR COMMUNITY-DWELLING OLDER ADULTS

**DOI:** 10.1093/geroni/igae098.2470

**Published:** 2024-12-31

**Authors:** Laurel Mertz, Jennifer Smith

**Affiliations:** Mather Institute, Evanston, Illinois, United States; Mather Institute, Evanston, Illinois, United States

## Abstract

Engagement in the arts has been shown to improve health and well-being in older adults. However, previous research suggests that some older adults may experience barriers to arts engagement, such as health concerns, cost, and lack of transport. One method to address these potential barriers for older adults is virtual arts programming. The purpose of this study was to determine older adults’ satisfaction with a six-week virtual art kit pilot program, as well as to examine how participation in the program affected participants’ health and well-being. The program involved participants receiving an art kit via mail with the supplies and instructions needed for each weekly project (e.g., scarf dying, affirmation weaving). Participation in weekly classes delivered via video conferencing and a social media group was encouraged. A total of 285 community-dwelling older adults completed both the pre- and post- surveys (96.1% female; 91.9% between the ages of 55 to 74). Analyses revealed high satisfaction with the program. Participants significantly improved from before to after the program on creativity, sense of community, purpose, stress, life satisfaction, and attitudes toward aging. There was no significant change in sense of mindfulness, loneliness, nor self-reported mental and physical health. This study provides evidence for the positive impact of a virtual art kit program on multiple aspects of well-being for older adults. Given the importance of accessibility in arts engagement, future research should continue to examine virtual arts programs as a method to enhance well-being.

